# Management of a Uterine Fibroid Originating From a Rudimentary Horn in a Patient With Mayer-Rokitansky-Küster-Hauser Syndrome: Report of a Rare Case

**DOI:** 10.7759/cureus.78598

**Published:** 2025-02-05

**Authors:** Elias Tsakos, Emmanouil M Xydias, Vasileios Emmanouil, Apostolos C Ziogas, Nikolaos Tsagias

**Affiliations:** 1 Department of Obstetrics and Gynecology, EmbryoClinic IVF, Thessaloniki, GRC; 2 Department of Medicine, School of Health Sciences, Democritus University of Thrace, Alexandroupoli, GRC; 3 Faculty of Medicine, School of Health Sciences, University of Thessaly, Larissa, GRC

**Keywords:** laparoscopic gynecological surgery, mayer-rokitansky-küster-hauser (mrkh) syndrome, ovarian cyst, uterine aplasia, uterine fibroid

## Abstract

Mayer-Rokitansky-Küster-Hauser (MRKH) syndrome involves complete or partial agenesis of the female reproductive tract, leading to impaired menstruation and reproduction, or even clinical symptoms in certain cases. The absence of an anatomically intact reproductive tract usually misleads clinicians to ignore the possibility that common gynecological conditions may develop in MRKH patients, which is a rare but plausible scenario. In the present report, we present the rare case of a 49-year-old MRKH patient with uterine fibroid developing from rudimentary uterine tissue, one of only a few similar instances reported in medical literature. The patient, scheduled to undergo oophorectomy due to a suspicious ovarian cyst and being perimenopausal, decided to undergo total laparoscopic en bloc excision of the ovaries, salpinges, uterine rudiments, fibroid, and ovarian cyst. The surgery was successful and the patient made a swift, uneventful recovery. This report adds to the limited knowledge of benign mesenchymal tumors arising from mere remnants of myometrium and affirms the need for vigilance during the care of MRKH patients.

## Introduction

Mayer-Rokitansky-Küster-Hauser (MRKH) syndrome is a type of congenital malformation characterized by impaired embryonic development of the Müllerian ducts, resulting in the complete or partial agenesis of the uterus and vagina, in individuals with a typical 46, XX karyotype [1]. Data on its prevalence varies from study to study from one in 4,000 to one in 20,000 [2], with the most recent and largest studies originating from Europe indicating an estimated prevalence of approximately one in 5,000 live female births [3] though prevalence for other parts of the world remains understudied.

Patients with MRKH syndrome, despite the malformation or absence of the uterus and/or vagina, typically present normal external genitalia, while also retaining normal ovarian growth and function. Due to the latter, the development of normal secondary sexual characteristics (e.g., breast development and pubic hair growth) remains typically unaffected [1,4], however, MRKH syndrome is expectedly associated with primary amenorrhea, making it the second most common cause of absent menstruation after Turner syndrome [1]. Approximately 90% of patients with MRKH syndrome have uterine remnants, which are often non-functional myometrial nodules, however, may be associated with chronic pelvic pain, requiring surgical intervention [4]. These remnants possess the histological features of the myometrium, namely, smooth muscle fibers and fibrous stroma, and are therefore in theory susceptible to the same gynecological conditions as the former, such as uterine fibroids.

Uterine fibroids, or leiomyomas, are the most prevalent benign pelvic tumors in women of reproductive age, affecting more than 70% of women globally [5]. Fibroids originate histologically from the fibromuscular tissue of the myometrium and are hormonally responsive tumors, influenced primarily by estrogen and progesterone. Their growth is facilitated by increased estrogen production, particularly in premenopausal women. In contrast to normal myometrial tissue, fibroids exhibit heightened expression of estrogen receptors (ER) and progesterone receptors (PR), rendering them more sensitive to steroid hormones. This hormonal hypersensitivity is a key factor in the pathogenesis of fibroids [6].

While MRKH patients may lack an anatomically intact uterus, they do frequently possess uterine remnants, which possess myometrial characteristics. This may lead to rare instances of leiomyomas growing from these remnants, following the same pathophysiological pathway as in a woman with an intact uterus [7]. In this report, we present an instance of this rare event occurring in an MRKH patient and its management.

## Case presentation

A 49-year-old woman with a BMI of 29.4 visited our clinic, following up on a recently diagnosed large ovarian cyst via ultrasound. From the patient’s medical history, of note was the diagnosis of MRKH syndrome at the age of 14 via laparoscopy, with hypoplastic uterus (two rudimentary horns), cervical aplasia, and normal vagina. The patient was obviously para 0 gravida 0 but had managed to have biological offspring (two twin boys) via gestational surrogacy. The patient reported no notable surgical history, with the exception of the aforementioned laparoscopy, and received no medication on a regular basis.

**Figure 1:**
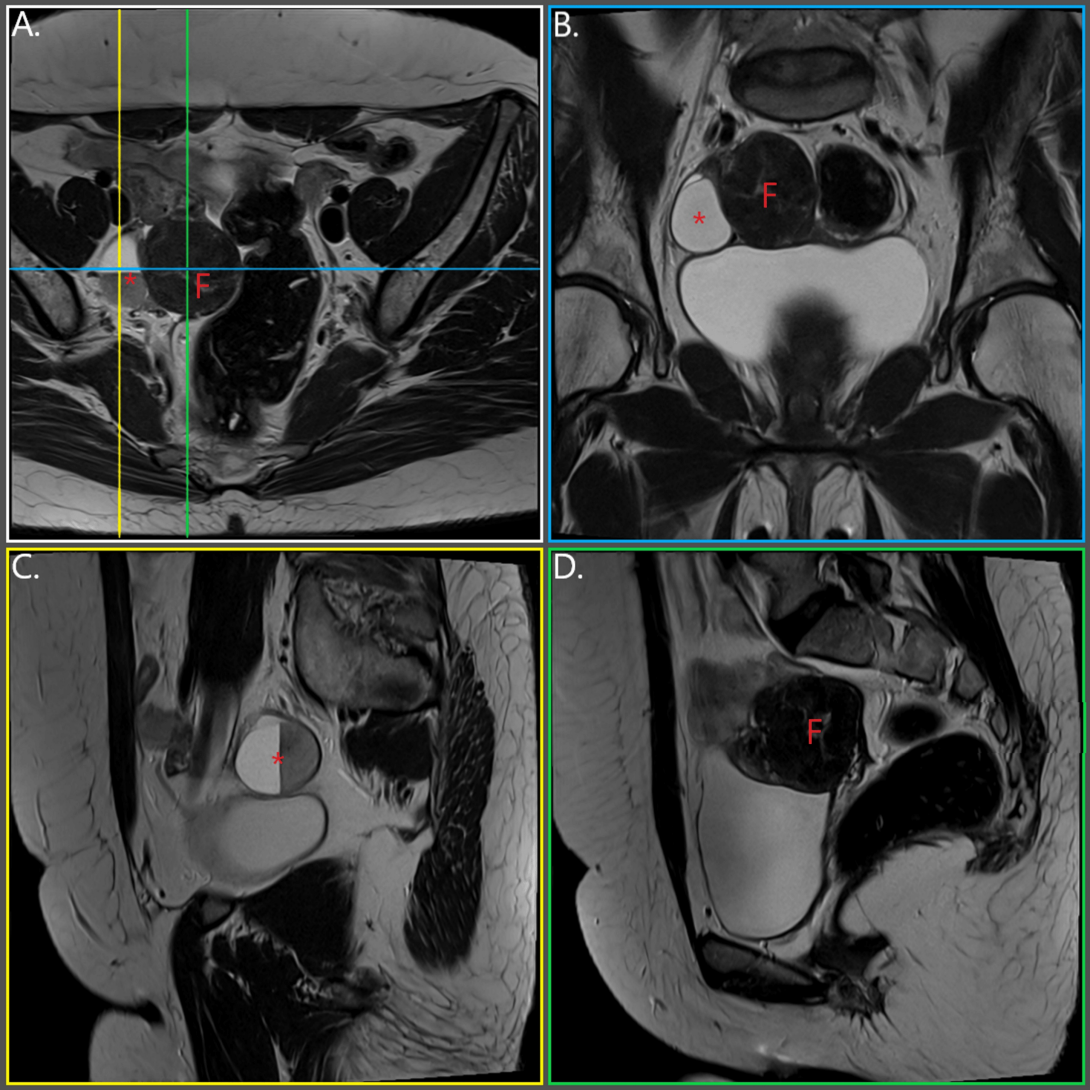
Preoperative pelvic and lower abdomen MRI scan, multiplanar reconstruction F: uterine fibroid; *: ovarian cyst (A) transverse plane; (B) coronal plane (blue); (C) longitudinal plane, level of ovarian cyst (yellow); (D) longitudinal plane, level of fibroid

Three months prior to her visit, during routine gynecological assessment, the patient was diagnosed with a sizeable cystic formation in the right ovary, of a diameter of approximately 3 cm, and a tumorous formation in the pelvis measuring approximately 4 cm in diameter. Ultrasonographic features mostly resembled mesenchymal derived mass; however, the differential diagnosis included pelvic abscess, ovarian or paraovarian mass, and hydrosalpinx. Given the suspicious characteristics of the ovarian cyst, the patient’s age, the distorted pelvic anatomy due to MRKH syndrome, and the presence of an unknown pelvic mass, she was instructed by her gynecologist to undergo a lower abdomen and pelvis MRI scan, which she did a month later (two months before her visit to our clinic). This scan confirmed the presence of a cystic formation in the anatomical region corresponding to the right ovary, measuring 3.8 x 3.4 cm, witan an apparent fluid-fluid level, attributed to a complex, hemorrhagic cyst. Additionally, the presence of a heterogenous formation resembling a uterine fibroid, measuring 4.4 x 3.8 cm was also confirmed in the pelvis (Figure 1).

Owing to the absence of previous medical records regarding the cyst and uterine fibroid and the suspicious characteristics of the cyst, the patient was advised to undergo surgical resection. The patient was uncertain and decided to seek a second opinion from a gynecological surgeon before considering her options, leading to her referral to our clinic two months later. After confirming the need for surgery, due to the patient’s own anxiety over similar conditions arising in the future and her perimenopausal status, she was offered the option of complete removal of both formations, uterine remnants, salpinges, and ovaries, with a histologic assessment of all specimens. Due to the patient’s age and desire for minimal scarring, the laparoscopic route was selected.

**Figure 2:**
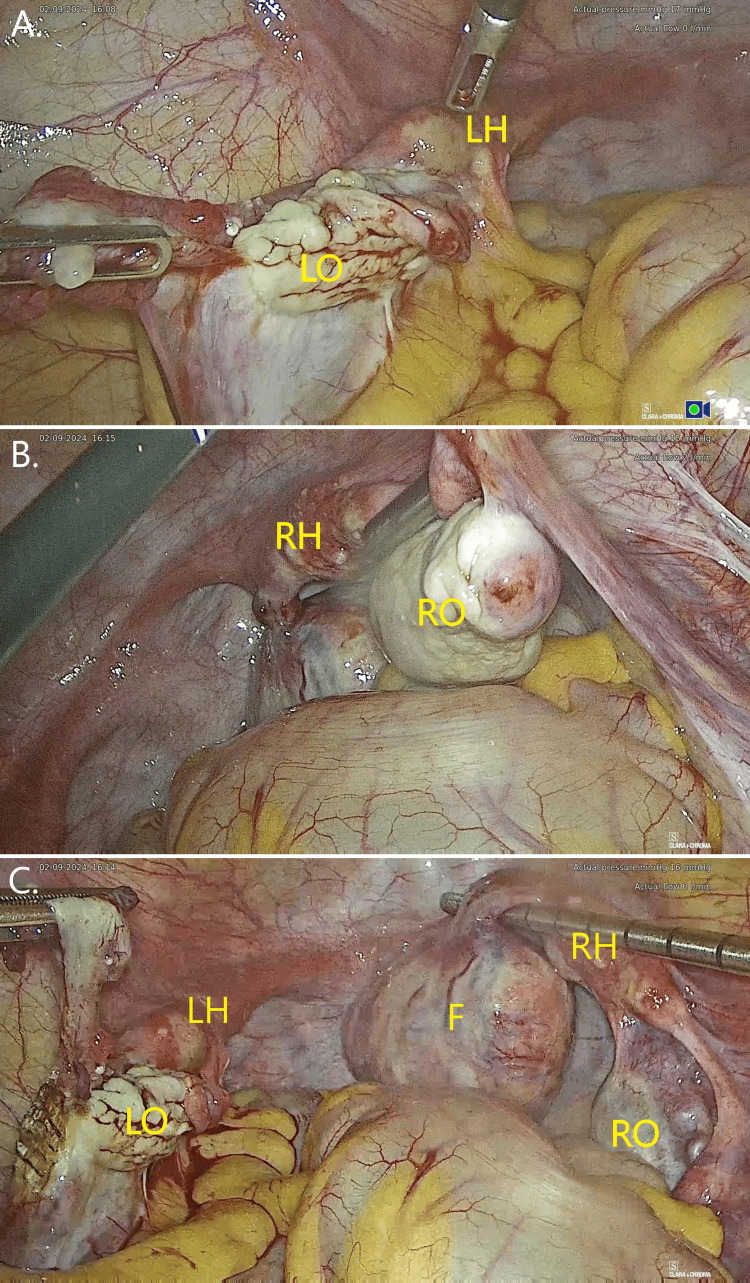
Laparoscopic inspection of the peritoneal cavity LO: left ovary; RO: right ovary; LH: left horn (in continuity with the left salpinx); RH: right horn (in continuity with the right salpinx); F: fibroid (A) Left ovary, salpinx, and rudimentary horn (less developed than contralateral one); (B) right ovary (with cyst), salpinx, and rudimentary horn; (C) overview or internal genitalia

The patient was operated on approximately one month after her initial presentation to our clinic. Following routine surgical preparation, a laparoscopy was performed. During the initial laparoscopic inspection, uterine aplasia was confirmed; absence of a cervix and normal corpus uteri, with only two rudimentary horns, and classified as U5aC4V0 as per the European Society of Human Reproduction and Embryology (ESHRE)/European Society for Gynaecological Endoscopy (ESGE) Classification System [8]. The right rudimentary horn was more developed than the left and was the one from which the uterine fibroid originated. The left horn was notably more aplastic, resembling residual myometrial tissue, which was in anatomical continuity with the salpinx. Both salpinges were present and normal, and the ovaries were present and atrophic bilaterally, a normal finding for the patient’s age. Additionally, the presence of a right ovarian cyst and a pedunculated subserosal uterine fibroid (The International Federation of Gynecology and Obstetrics (FIGO) 7) was confirmed (Figure 2).

**Figure 3:**
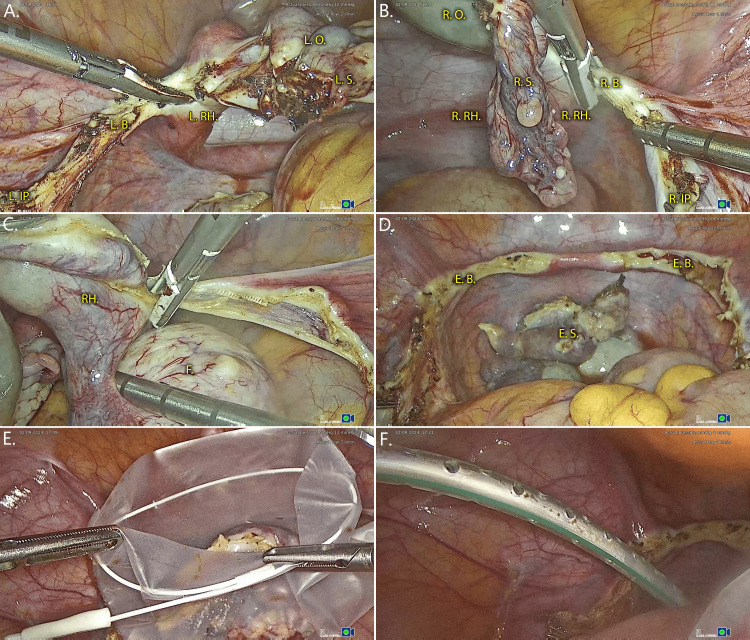
Laparoscopic resection of rudimentary uterine horns and salpingo-oophorectomy L. IP.: left infundibulopelvic ligament, clipped and cut (mostly out of frame); L.B.: left broad ligament; L.RH.: left rudimentary horn; L.S.: left salpinx; L. O.: left ovary; R. IP.: right infundibulopelvic ligament, clipped and cut; R.B.: right broad ligament; R.RH.: right rudimentary horn; R.S.: right salpinx; R. O.: right ovary; RH.: rudimentary horn; F: fibroid; E.B.: excised broad ligament; E.S.: excised specimens (A) Mobilization and en bloc excision of left ovary, salpinx, and rudimentary horn; (B) mobilization and en bloc excision of right ovary, ovarian cyst, salpinx, and rudimentary horn; (C) fibroid is resected en bloc with the right rudimentary horn; (D) overview of peritoneal cavity after resection; (E) placement of specimens in surgical bags and extraction; (F) abdominal drain placement

The surgery proceeded under general anesthesia. Infundibulopelvic ligaments were desiccated via cautery and surgical clips were placed prior to their resection. The rest of the ovary, salpinx, and rudimentary horn were mobilized via resection of the broad ligament remnants on the lateral pelvic walls bilaterally and were removed en bloc with the ovarian cyst and fibroid. After proper hemostasis and irrigation, the specimens were placed in surgical bags and removed via the port incision, using electric motor morcellation. Finally, an abdominal drain was placed, and the port incisions were sutured in two layers. Important surgical highlights are visualized in Figure 3. Total operative time was two hours and 30 minutes, with minor blood loss and a recorded 1g/dl drop in mean Hb concentration. The patient had no early postoperative complications and no notable symptoms, except for mild fatigue during the first 24 hours post-operatively, which completely subsided on the second day of stay. The patient received routine prophylactic postoperative antibiotics (amoxicillin and clavulanic acid (825 mg/125 mg; twice per day for four days) and anticoagulants (enoxaparin anti-Xa: 4,000 IU; one injection daily for four days), remained hospitalized for two days, and was discharged without any complications. Postoperative follow-up at 15 and 30 days revealed no late-onset complications, and the patient reported a swift and uneventful recovery. Macroscopic assessment of derived specimens by a histopathologist revealed two whitish formations of 6 and 2 cm, respectively, the latter within one of the uterine remnants. Microscopic assessment of these formations showed intertwining spindle-like cells, with eosinophilic cytoplasm, elongated nuclei and small nucleoli, mild cellular proliferation and absence of karyokinesis, cellular atypia, and necrosis, confirming the presence of fibroids; one known and the other within the uterine horn. The suspicious ovarian cyst was confirmed to be an endometrioma no evidence of malignancy was noted in any of the specimens sent for study.

## Discussion

In this report, we presented the management of a rare case of leiomyoma arising in the uterine remnants of an MRKH syndrome patient. Although rare, similar cases have been reported before. The first ever recorded instance of a fibroid in a patient with MRKH syndrome was published in 1977, with more recent reports also available [9]. In most cases, the initial assessment typically involves ultrasonography, which may be performed transabdominally or transvaginally [10-14]. Additional imaging studies, as part of the differential diagnosis and to ascertain the topography, may include magnetic resonance imaging (MRI) and computed tomography (CT). This was similar to our experience in the present case, where initial diagnosis was performed via transvaginal ultrasound, and a follow-up assessment was performed via MRI, although this was mostly in the context of the ovarian cyst investigation.

Interestingly, two of the reported cases were managed as emergencies due to acute abdominal symptoms, resulting from the torsion of the uterine remnant leiomyomas [11,14]. This is in contrast to our case, where the diagnosis of the leiomyoma was incidental and its management secondary to the more clinically urgent suspicious ovarian cyst. This highlights the potentially elusive nature of gynecological pathology occurring in such women and the need for constant vigilance on behalf of the gynecologist. In terms of the therapeutic approach, the primary strategy employed by most investigators is the excision of the mass, which may be performed laparoscopically or via laparotomy with a midline vertical incision. The treatment options then range from exclusive excision of the mass [13,15] to the unilateral [12,14] or even bilateral [10] removal of uterine remnants, or potentially the adnexal structures, especially in cases of elevated clinical suspicion of ovarian malignancy [16]. The strategy followed in our case falls under the latter path, whereby both uterine remnants, adnexa, and fibroids were removed, given the patient’s age, diagnosis of a clinically suspicious cyst, and preference.

MRKH syndrome is a complex and diverse clinical condition, which may affect multiple aspects of the patient’s health. It may be confined to the Müllerian ducts, resulting in the absence or underdevelopment of the uterus and vagina without the involvement of other organ systems (MRKH type I: isolated or Rokitansky sequence), or it may also involve the renal system and somite structure, leading to more severe systemic defects (MRKH type II: MÜllerian duct aplasia, renal aplasia, cervicothoracic somite dysplasia (MURCS) association), thus further complicating its management [17]. Even in its simpler manifestation, MRKH remains a clinical challenge. While anatomically compromised, the internal genitalia are susceptible to the same ailments as those of the general female population, as was the case in the patient presented in this report. More than 90% of individuals with MRKH syndrome have uterine remnants, typically myometrial nodules that lack endometrial differentiation [4]. These remnants may cause chronic pelvic pain on their own or be complicated by other, more common gynecologic conditions, such as uterine fibroids [4]. Pelvic MRI remains the gold standard for diagnosis, providing a detailed assessment of the uterine buds and the extent of uterovaginal agenesis [4], and surgical removal of the rudimentary uterus is the most effective option for symptomatic cases [15]. In addition to clinical symptoms, a psychosocial component is highly pronounced in such patients, for whom the issue of childlessness and self-consciousness of their body image may profoundly affect their lives. MRKH patients frequently experience significant distress related to infertility, sexual identity, and the fear of coital dysfunction. As a result, there is an elevated incidence of anxiety and depression among affected women [1]. This component was not as pronounced in the present case, given the advanced reproductive age of the patient and the completion of her family plan years prior; however, practitioners should remain conscious of the psychosocial implications of MRKH for their younger patients in particular.

The management of MRKH syndrome, especially when complicated by concomitant conventional gynecological conditions, such as uterine fibroids, requires a multidisciplinary approach to address all the medical and psychological aspects of the condition. Symptoms may be present and guide diagnosis; however, as the present case demonstrates, clinical manifestations may be completely absent as well, rendering frequent, thorough gynecological assessment the cornerstone of timely diagnosis. Treatment is primarily surgical and preferably minimally invasive, such as laparoscopic and robotic surgery, which minimize patient trauma and produce a more aesthetically pleasing result and are becoming increasingly popular in gynecological surgery [18]. Treatment options for MRKH-related sexual dysfunction include vaginal dilation therapy to address vaginal agenesis and the creation of a neovagina [19], as well as psychological support to address MRKH-related anxiety and depression. Finally, reproductive options, though admittedly limited, do exist. Excluding adoption, the only currently available option for these women to have biological offspring is gestational surrogacy, a highly successful program applied throughout the world, which however does come with certain ethical implications [20]. An experimental alternative, uterine transplantation surgery promises to eliminate these ethical issues and to allow these women to experience pregnancy themselves; however, further research is required for its wider application [21].

## Conclusions

MRKH syndrome represents a clinical condition with a significant impact on patients’ lives. In rare occasions, residual uterine remnants may unexpectedly give rise to uterine fibroids and other gynecological conditions, which, as highlighted by the present case, may be asymptomatic and remain undiagnosed for long periods of time. Heightened clinical awareness and frequent monitoring via physical examination and ultrasound are key to the timely diagnosis of underlying conditions. Treatment is primarily minimally invasive and benefits from a multidisciplinary approach, including attention to psychosocial parameters of the condition and sexual dysfunction. Thus, this holistic approach is essential in ensuring patient safety, addressing clinical symptoms, improving quality of life, and offering effective sexual and reproductive health therapeutic options.
